# Supplementary material for: A combined computational and experimental strategy identifies mutations conferring resistance to drugs targeting the BCR-ABL fusion protein
Source: Commun Biol. 2020 Jan 9;3:18. doi: 10.1038/s42003-019-0743-5 (PMC6952392; doi:10.1038/s42003-019-0743-5)
Supplement: Supplementary file 1 — Supplementary Information [file 42003_2019_743_MOESM1_ESM.pdf]

Supplementary Information

I. Distribution of calculated drug resistance mutations

The drug resistance mutations of the three drugs (imatinib, nilotinib and dasatinib) were predicted using EVER. The evolution process was carried out independently for three times and the frequently occurring mutants were analyzed.

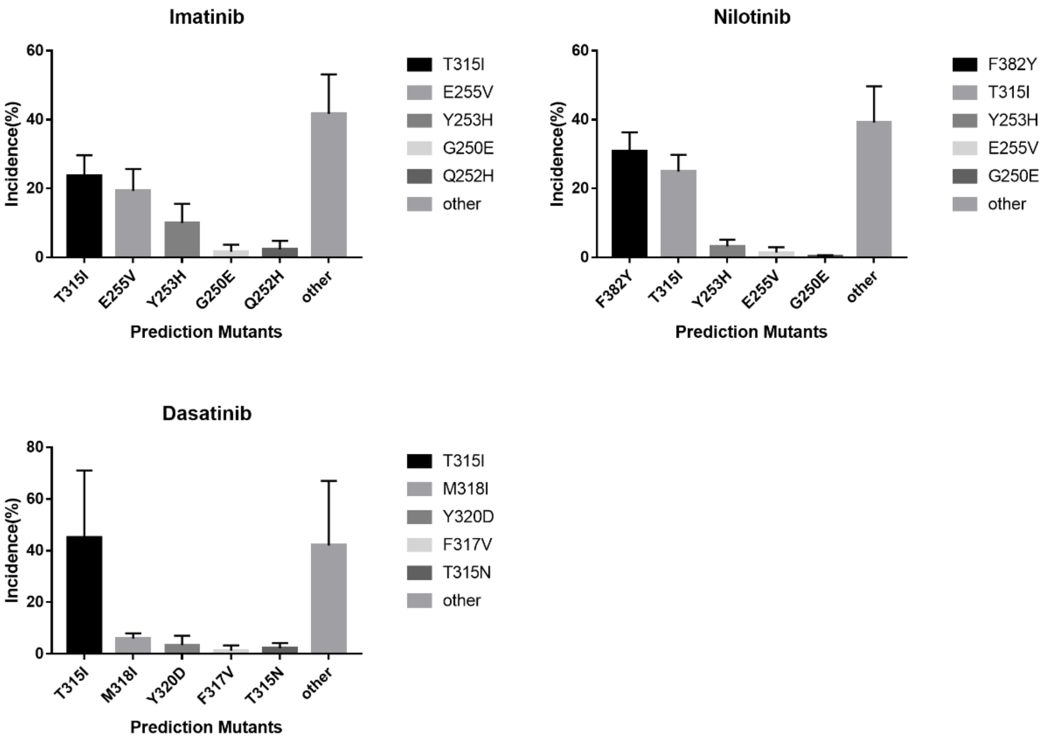

Supplementary Figure 1. Predicted drug resistance mutation types of imatinib, nilotinib and dasatinib. The results were of statistical convergence. The T315I mutation is dominant for each of the three drugs. However, the simulation results for nilotinib show that F382Y also has obvious advantages. And the simulation results for dasatinib, M318I and Y320D, these two types of mutations also occur frequently. For imatinib, the incidence of T315I, E255V, Y253H, G250E, Q252H and other is 23.8±6.0, 19.3±6.4, 10.0 ± 5.6, 1.7 ± 2.1, 2.3 ± 2.4, and 41.7 ± 11.5, respectively. For nilotinib, the incidence of F382Y, T315I, Y253H, E255V, G250E and other is 30.9±5.4, 25.0±4.9, 3.2±2.0, 1.5±1.5, 0.3±0.4 and 39.2 ±10.6, respectively. For dasatinib, the incidence of T315I, M318I, Y320D, F317V, T315N and other is 45.1±25.9, 6.0±2.0, 3.4±3.6, 1.2

±2.1, 2.2±1.9 and 42.1±25.0, respectively. n=3 independent simulations.

II. Protein heat denaturation

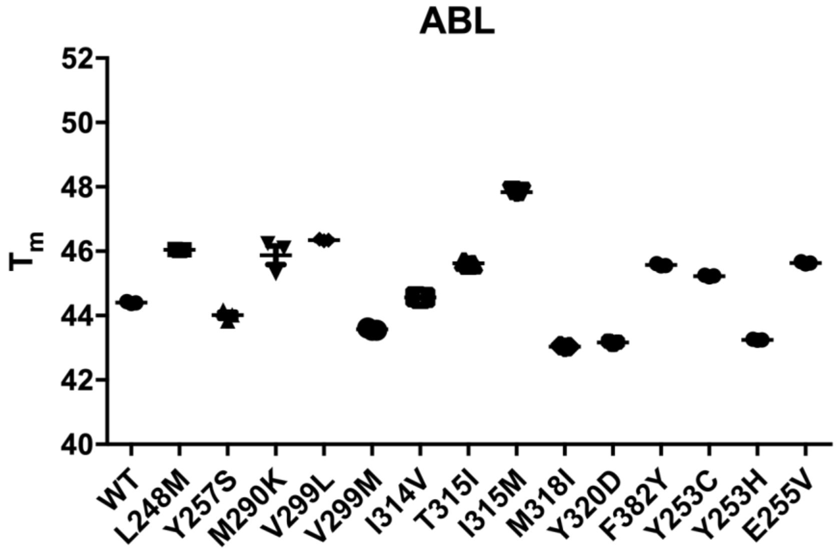

Supplementary Figure 2. Determination of ABL heat denaturation temperature by nanoDSF. The denaturation temperature of the kinase did not change significantly.

III. Protein secondary structure measurement

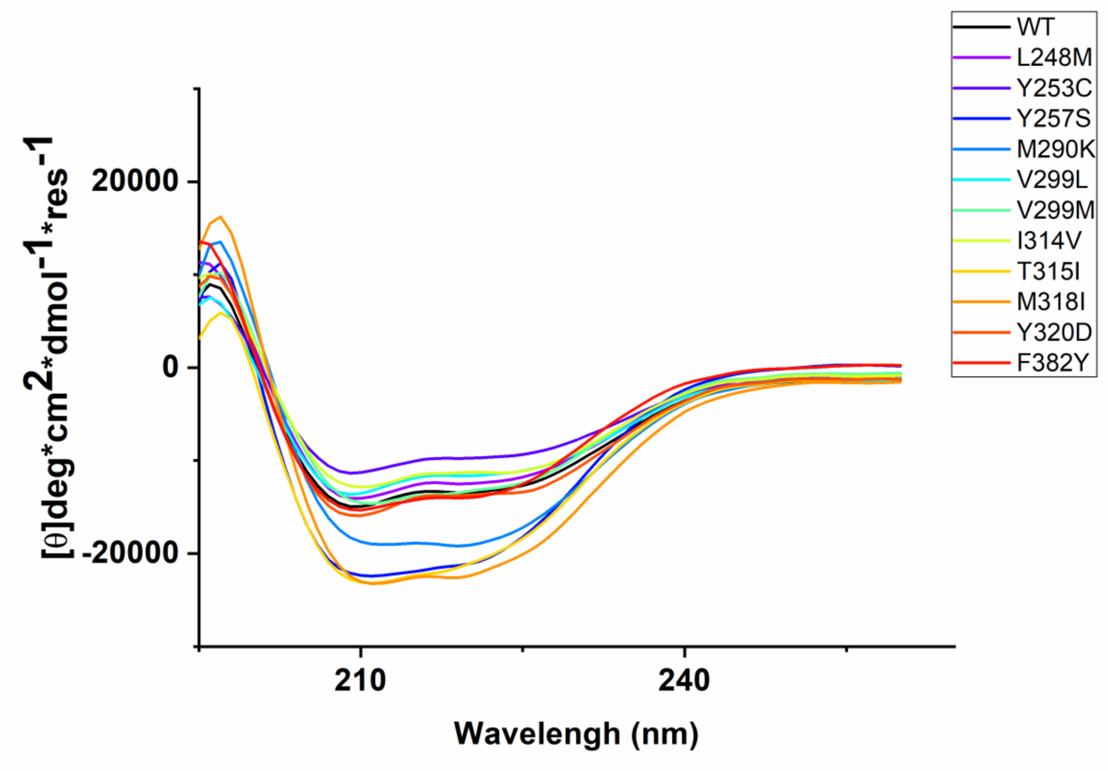

Supplementary Figure 3. Protein secondary structure measurement using circular dichroism. Wild type ABL kinase is black line. Almost all mutants have similar curves.

IV. Direct binding measurement of ABL kinase and drugs by ITC and MST

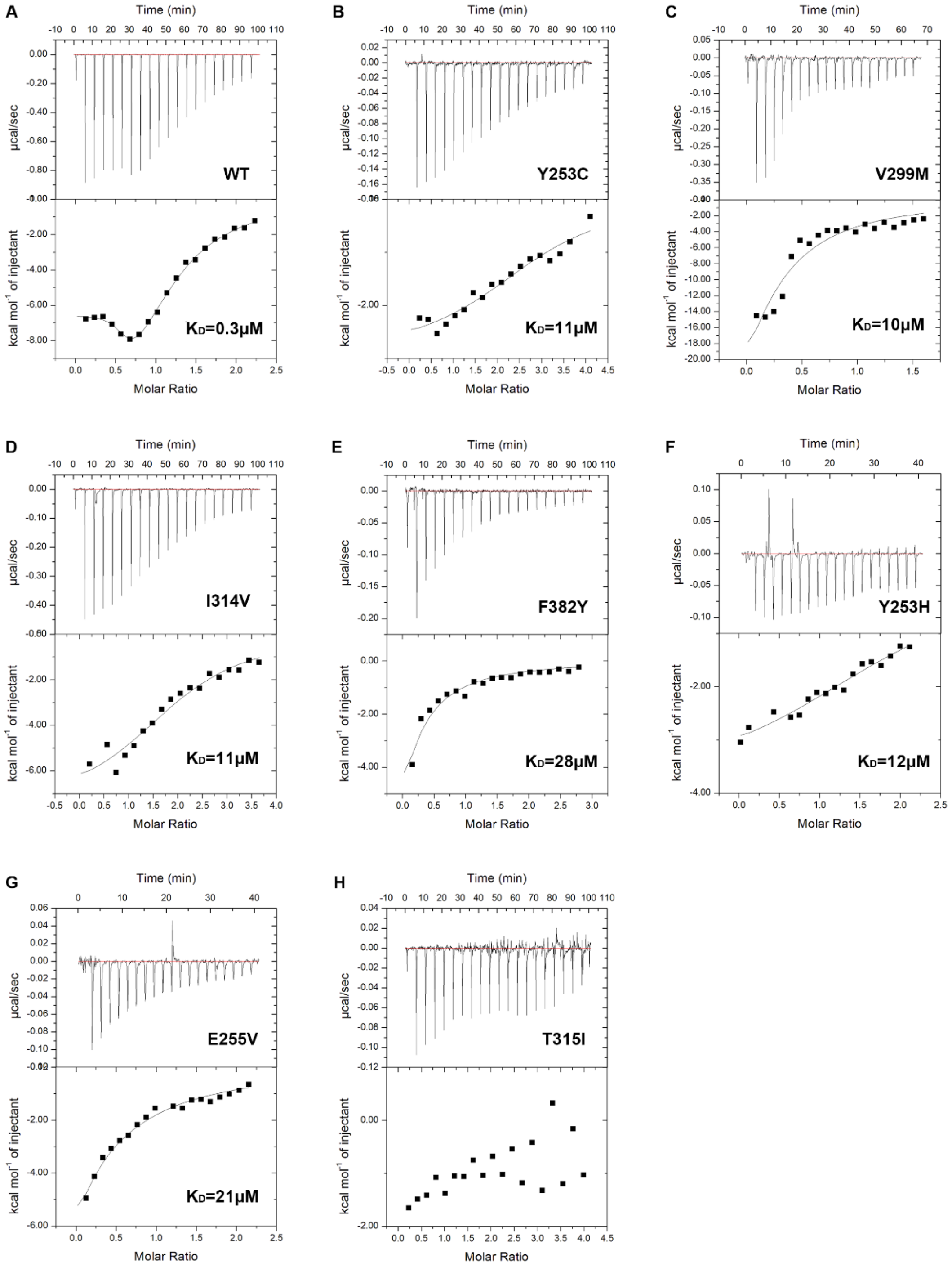

Supplementary Figure 4. The dissociation constant (K_D) measurement of imatinib and ABL kinase/mutants using ITC. The binding ability of the protein mutants and imatinib was significantly decreased. The three clinical resistance mutations tested are Y253H, E255V and T315I. **(A)** The K_D value determined by ITC for imatinib is 0.3±0.1μM. **(B)** Y253C + imatinib, K_D = 11.4±1.7 μM. **(C)** V299M + imatinib, K_D = 10.5±0.8 μM. **(D)**

I314V + imatinib, K_D = 11.1±4.4μM. **(E)** F382Y + imatinib, K_D = 28.4±8.8 μM. **(F)** Y253H + imatinib, K_D = 11.6±1.7 μM. **(G)** E255V + imatinib, K_D = 21.4±4.7 μM. **(H)** T315I's K_D > 128μM.

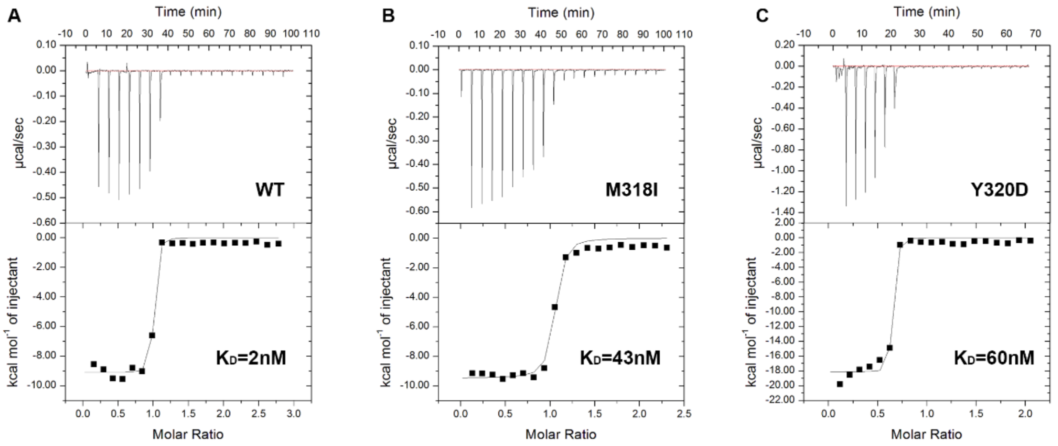

Supplementary Figure 5. K_D measurement of dasatinib and ABL kinase/mutants using ITC. The binding of dasatinib it stronger than the first-generation drug, imatinib. **(A)** the K_D for dasatinib as determined by ITC, is 2.0±0.2 nM. **(B)** M318I + dasatinib, K_D = 43.0±15.0 nM. **(C)** Y320D + dasatinib, K_D = 60±23 nM. K_D of the T315I mutation, the most common type of mutation found in the clinic, was too low to be quantitatively measured.

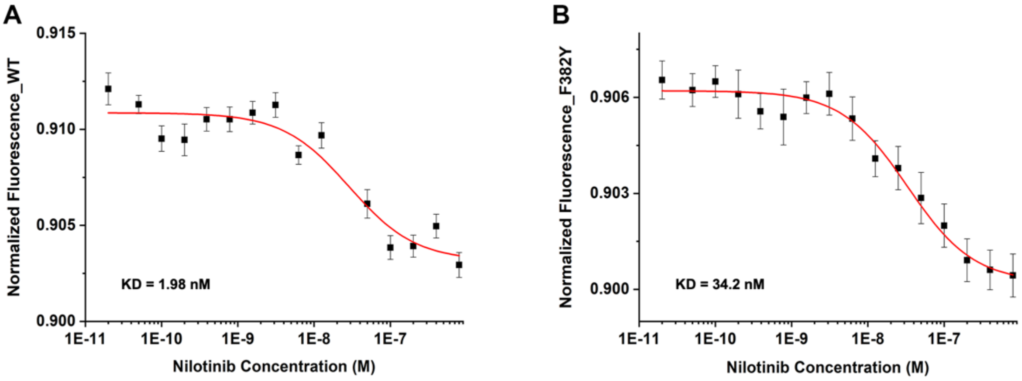

Supplementary Figure 6. K_D measurement of nilotinib and ABL kinase/mutants using MST. The binding ability of the mutated protein and the nilotinib was significantly decreased. **(A)** WT + nilotinib, K_D = 2.0±0.6 nM. **(B)** F382Y + nilotinib, K_D = 34.2± 6.0 nM. n=3 biologically independent experiments.

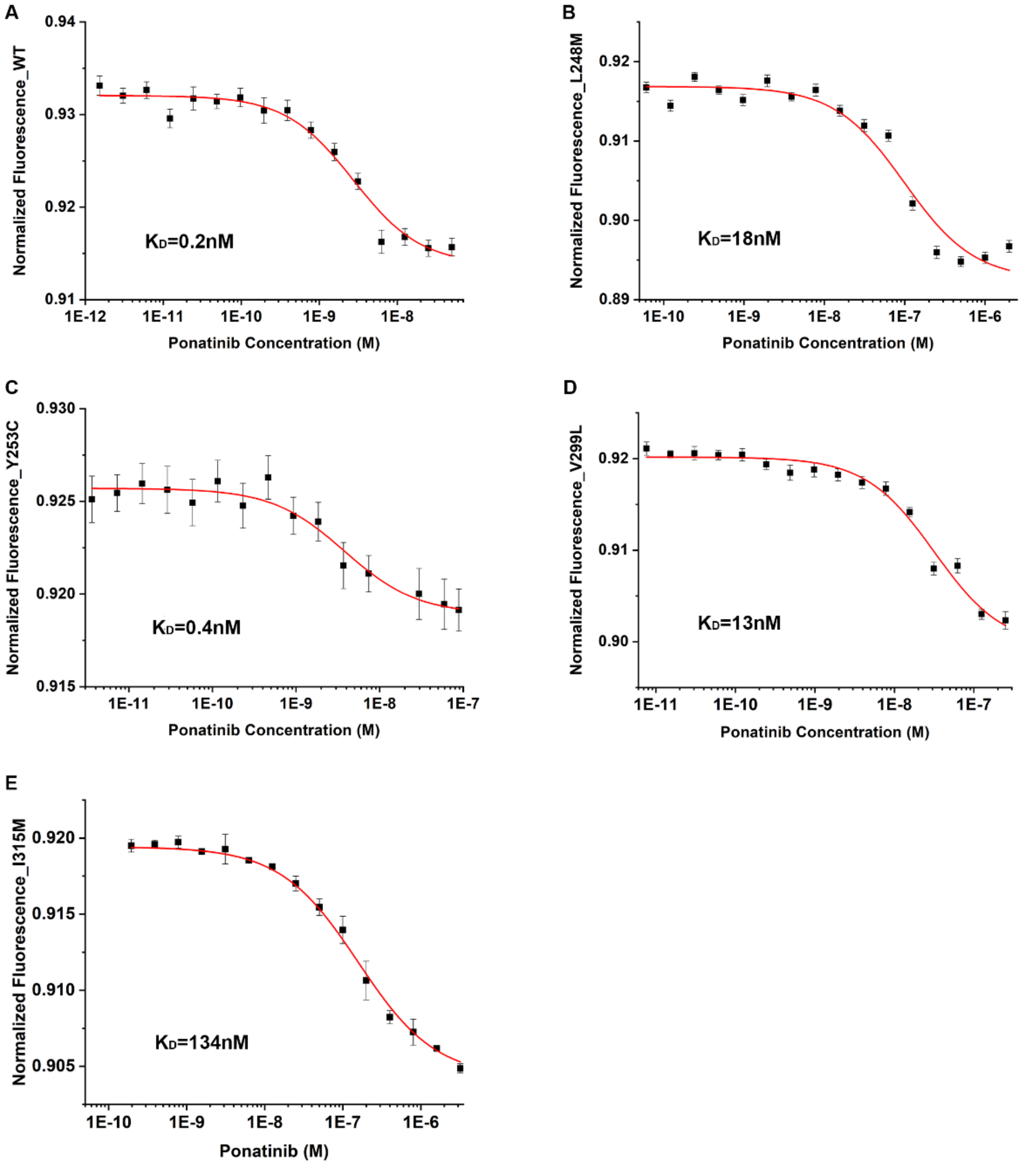

Supplementary Figure 7. K_D measurement of ponatinib and ABL kinase/mutants using MST. I315M is a drug-resistance mutation found in the clinic. **(A)** WT + ponatinib, K_D = 0.2±0.1 nM. **(B)** L248M + ponatinib, K_D = 18±2.7 nM. **(C)** Y253C + ponatinib, K_D = 0.4±0.1 nM. **(D)** V299L + ponatinib, K_D = 13.0±6.0 nM. **(E)** I315M +ponatinib, K_D = 134. 0±9.0 nM. n=3 biologically independent experiments.

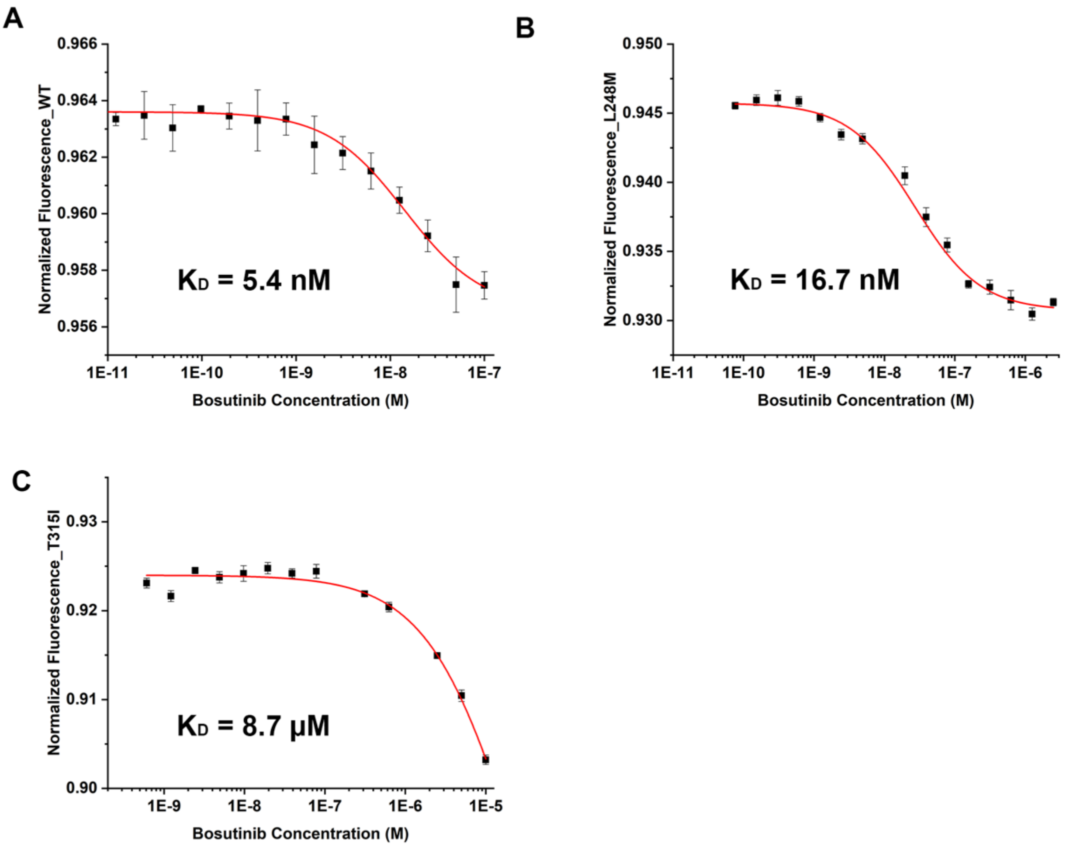

Supplementary Figure 8. K_D measurement of bosutinib and ABL kinase/mutants using MST. I315M is a drug-resistance mutation found in the clinic. **(A)** WT + bosutinib, K_D = 5.4±1. 2 nM. **(B)** L248M + bosutinib, K_D = 16.7±4.0 nM. **(C)** T315I + bosutinib, K_D = 8.7±0. 2 μM. n=3 biologically independent experiments.

V. The half-inhibition constant measurement of drugs and ABL kinase/mutants

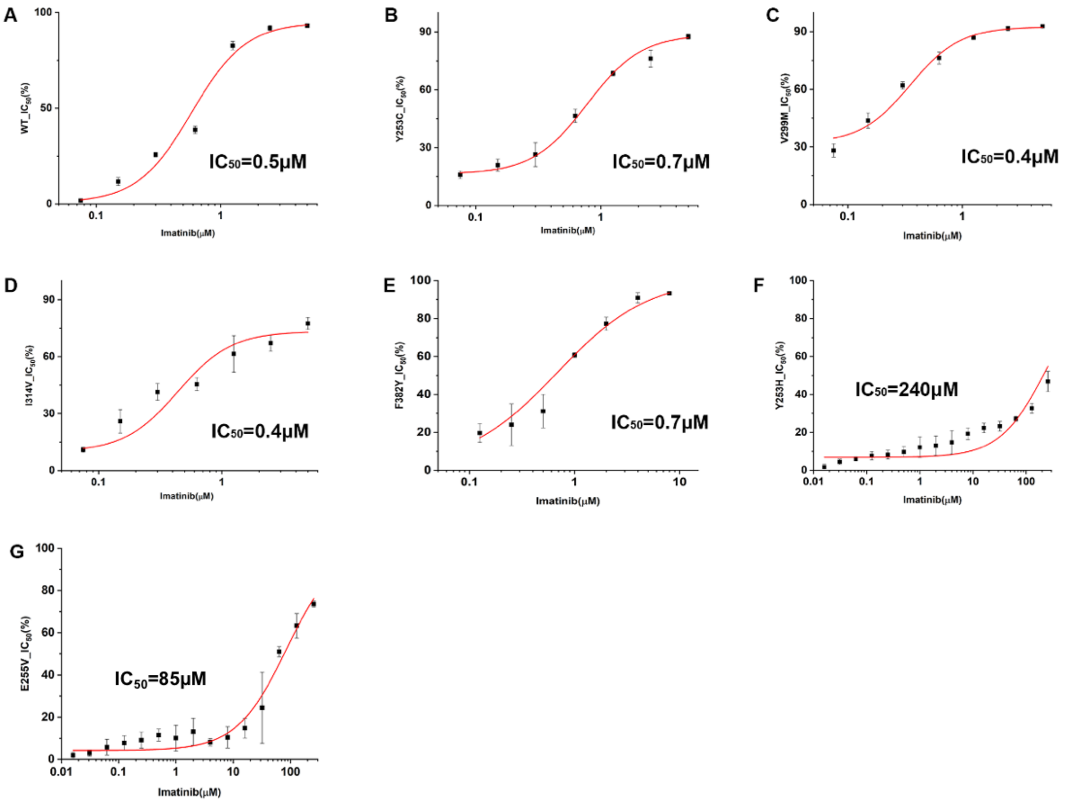

Supplementary Figure 9. IC_{50} of imatinib to wt and mutant ABL kinase. Unlike the change in binding ability, the IC_{50} of imatinib and mutants did not change obviously. Inhibition can be achieved by increasing the drug concentration. The clinically occurring resistance mutations of Y253H, E255V and T315I have a great influence on IC_{50}. It is difficult to produce an inhibitory effect under safe blood concentration conditions. The IC_{50} of the T315I cannot be measured due to its high resistance. **(A)** WT + imatinib, IC_{50} = 0.5±0.1 μM. **(B)** Y253C + imatinib, IC_{50} = 0.7±0.1 μM. **(C)** V299M + imatinib, IC_{50} = 0.3±0.1 μM. **(D)** I314V + imatinib, IC_{50} = 0.4±0.1 μM. **(E)** F382Y + imatinib, IC_{50} = 0.7±0.1 μM. **(F)** Y253H + imatinib, IC_{50} = 240.0±36.0 μM. **(G)** E255V + imatinib, IC_{50} = 84.8±7.2 μM. n=3 biologically independent experiments.

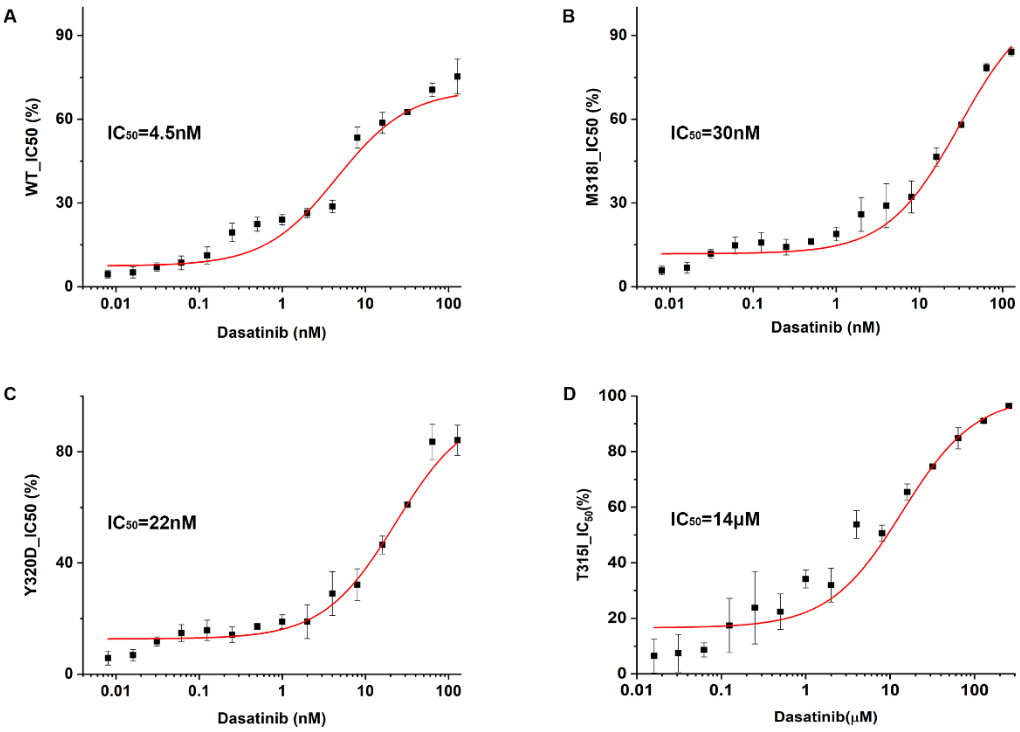

Supplementary Figure 10. IC_{50} of dasatinib to wt and mutant ABL kinase. **(A)** WT + dasatinib, IC_{50} = 4.5±1.1 nM. **(B)** M318I + dasatinib, IC_{50} = 30.0±5.0 nM. **(C)** Y320D + dasatinib, IC_{50} = 22.0±8.0 nM. **(D)** T315I + dasatinib, IC_{50} = 14.0±0.9 μM. n=3 biologically independent experiments.

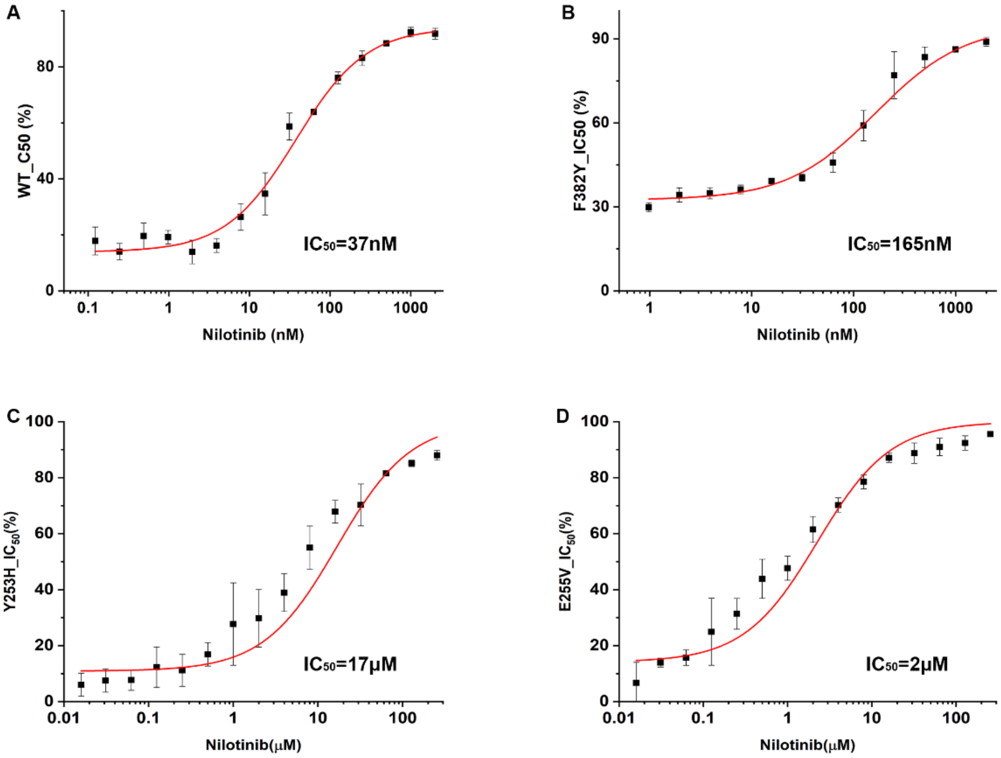

Supplementary Figure 11. IC_{50} of nilotinib to wt and mutant ABL kinase. The IC_{50} of T315I and nilotinib could not be measured due to weak inhibition. **(A)** WT + nilotinib, IC_{50} = 37.0±2.3 nM. **(B)** F382Y + dasatinib, IC_{50} = 165±33 nM. **(C)** Y253H + nilotinib, IC_{50} = 17.0±1.1 μM. **(D)** E255V + nilotinib, IC_{50} = 2.3±0.4 μM. n=3 biologically independent experiments.

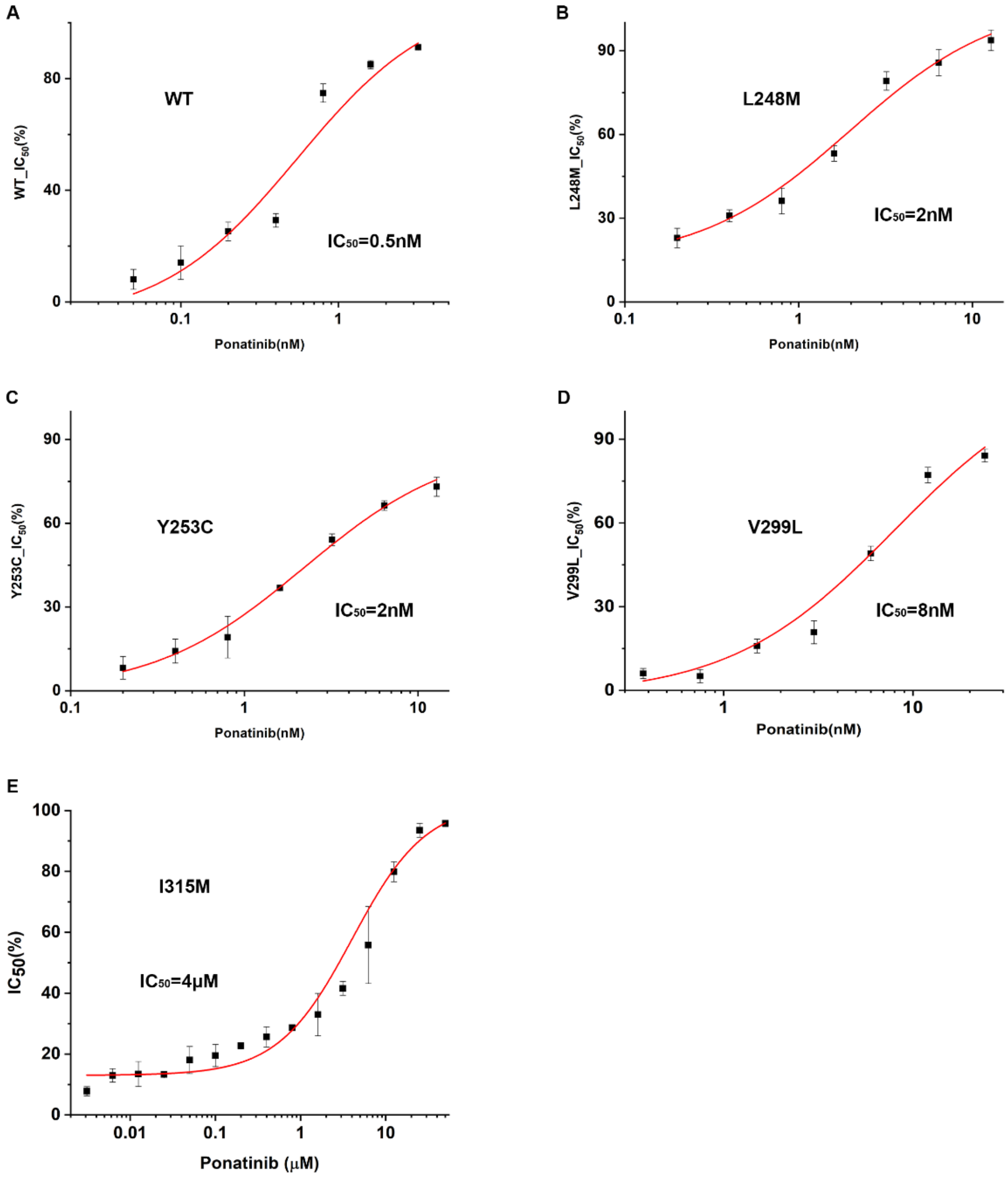

Supplementary Figure 12. IC_{50} of ponatinib to wt and mutant ABL kinase. **(A)** WT + ponatinib, IC_{50} = 0.5±0.3 nM. **(B)** L248M + ponatinib, IC_{50} = 2.0±0.6 nM. **(C)** Y253C + ponatinib, IC_{50} = 2.2±0.3 nM. **(D)** V299L + ponatinib, IC_{50} = 8.0±3.0 nM. **(E)** I315M + ponatinib, IC_{50} = 4.0±0.6 μM. n=3 biologically independent experiments.

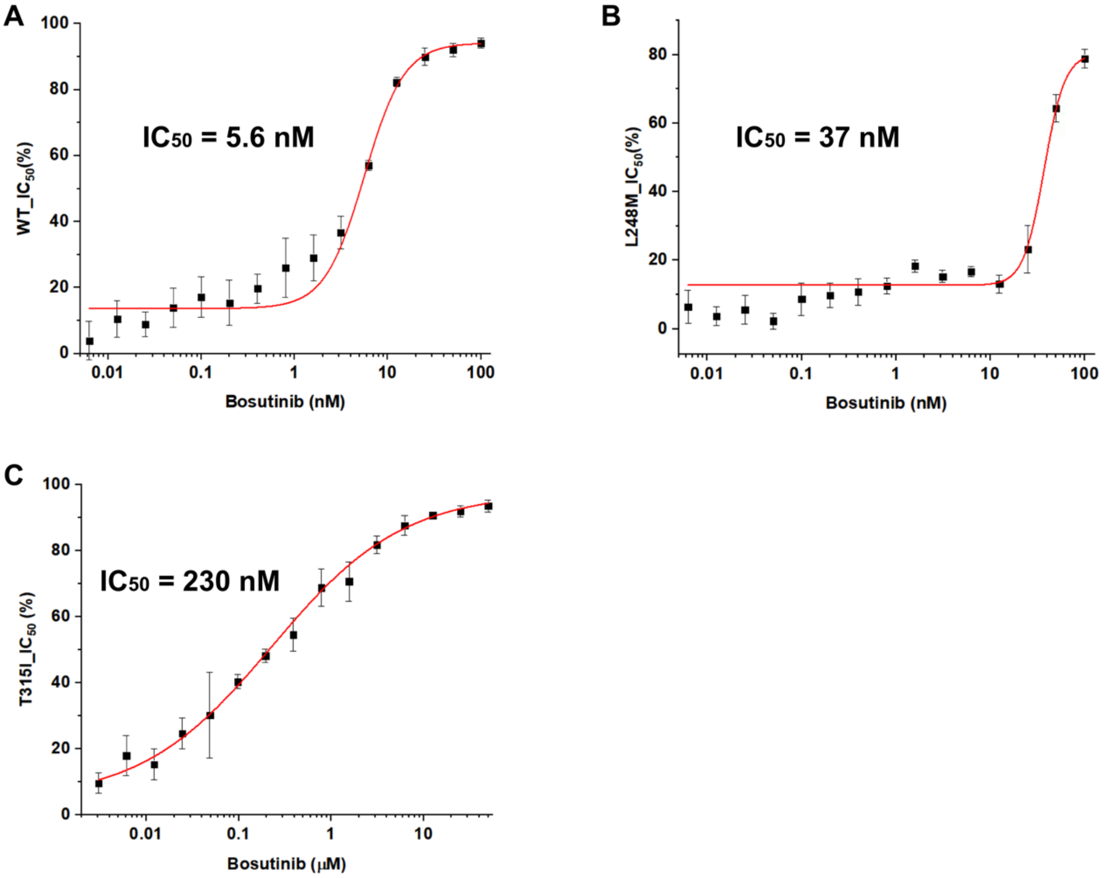

Supplementary Figure 13. IC_{50} of bosutinib to wt and mutant ABL kinase. **(A)** WT + bosutinib, IC_{50} = 5.6±0.3 nM. **(B)** L248M + bosutinib, IC_{50} = 37.0±7.7 nM. **(C)** T315I + bosutinib, IC_{50} = 230.0±25.0 nM. n=3 biologically independent experiments.

VI. Measuring the catalytic parameters for ABL kinase/mutants using single injection ITC method

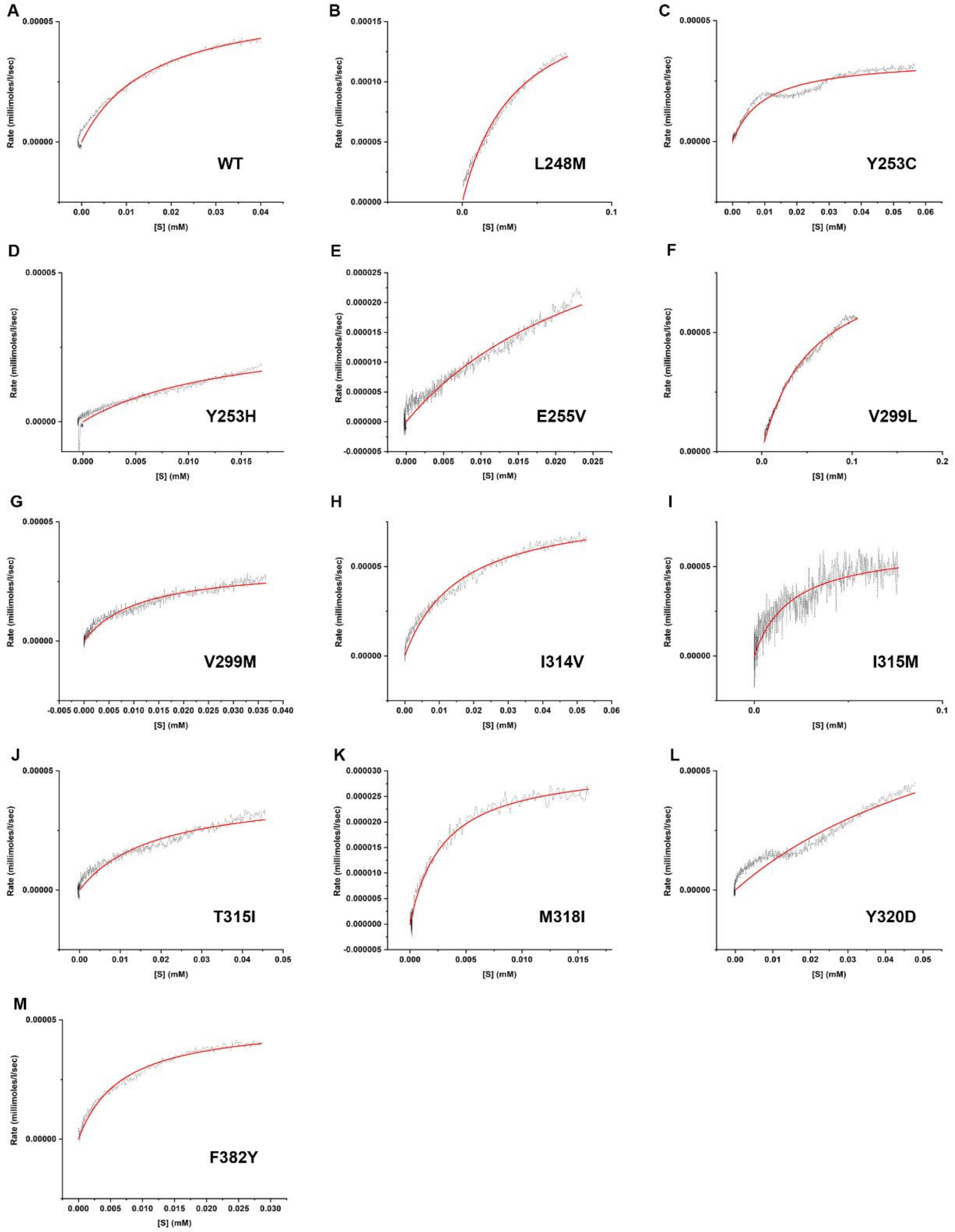

Supplementary Figure 14. ITC single injection measurement for ABL kinase and mutants. The exothermic process of the enzymatic reaction is determined by ITC. The enzyme kinetic constants were calculated according to the single injection method. A-N are diagrams of the exothermic process of different mutants. Enzyme kinetic constants were obtained by fitting to the OriginLab software.

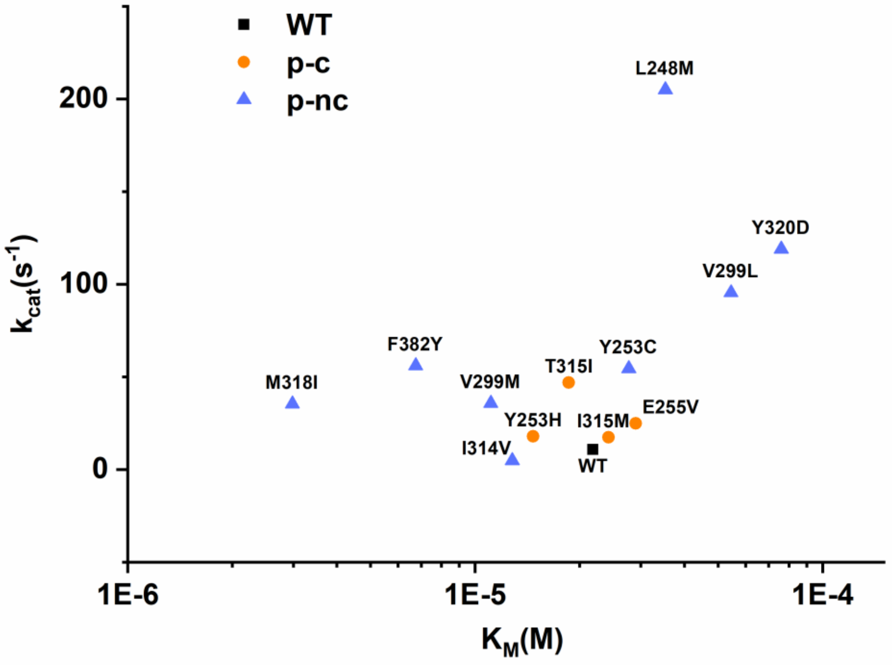

Supplementary Figure 15. Distribution of ABL kinase catalytic parameters for wild type, p-c and p-nc mutants. The p-c mutants have catalytic potency similar to wt protein, while the p-nc mutants have either lowered K_M or elevated k_{cat}.

VII. Distribution of calculated ponatinib resistance mutations

Since ponatinib is a drug designed for T315I, we used two different sequences for prediction. One is the wild type sequence of T315. The other is the sequence of I315. The sequence prediction results based on I315 are relatively stable to result in I315M, which is a clinically resistance mutation. However, the sequence results based on T315 are diversified, and no obvious dominant mutations appear. Therefore, the prediction results of each generation of resistance mutations are counted.

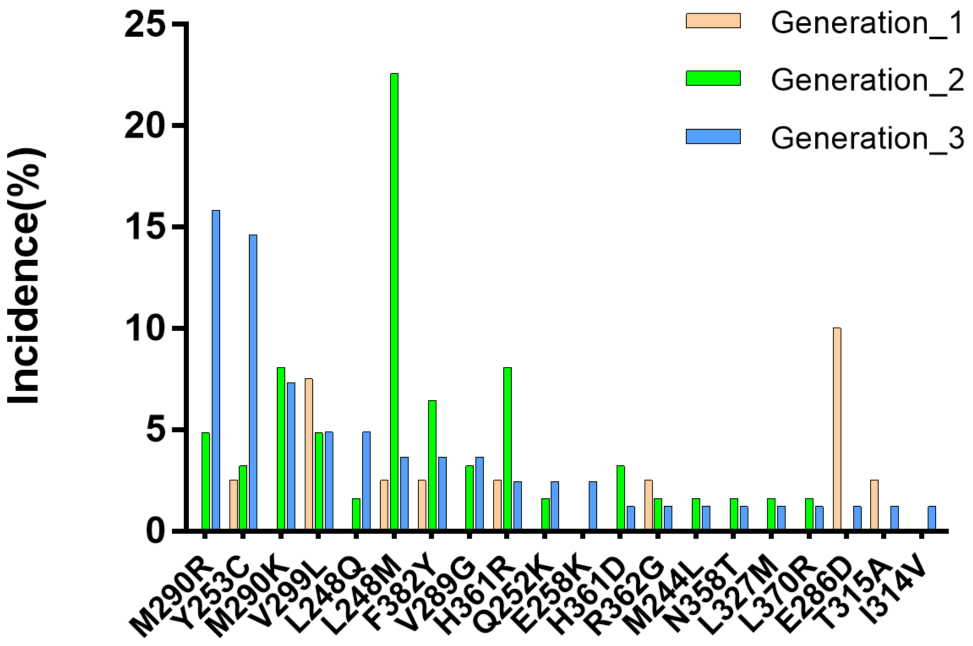

Supplementary Figure 16. EVER predicted drug resistance mutations for ponatinib with the wild type sequence (T315)

VIII. Data summary

Supplementary Table 1. Summary of experimental data for drug binding, enzyme inhibition and enzyme catalysis of various drugs and ABL kinase/mutants

Drugs	Types	Proteins	K_D (nM)	IC_{50} (nM)	k_{cat} (s^{-1})	$K_{M(ATP)}$ (μM)
imatinib	WT		316.0±29.5	584.0±77.3	10.8±0.2	21.8±0.8
	p-c[1]	Y253H	11671±1745	240000±36086	19.5±0.7	19.2±1.1
		E255V	21490±4691	84862±7158	24.8±1.0	29.0±1.7
		T315I	>128000	>468000	46.9±0.8	28.6±0.7
	p-nc[2]	Y253C	11417±1387	774±39	54.4±0.7	27.7±0.8
		V299M	10530±826	349±55	35.7±0.5	11.1±0.4
		I314V	11125±4403	440±108	4.78±0.1	12.8±0.6
		F382Y	28400±8845	685±127	56.0±0.5	6.75±0.2
nilotinib	WT		2.0±0.6	37.0±2.3	10.8±0.2	21.8±0.8
	p-c[1]	Y253H	1100±264	16844±1118	19.5±0.7	19.2±1.1
		E255V	1690±314	2259±364	24.8±1.0	29.0±1.7
		T315I	>128000	>273000	46.9±0.8	28.6±0.7
	p-nc[2]	F382Y	34.2±6.0	165±33	56.0±0.5	6.75±0.2
dasatinib	WT		2.0±0.2	4.5±1.1	10.8±0.2	21.8±0.8
	p-c[1]	T315I	>2560	13896±914	46.9±0.8	28.6±0.7
	p-nc[2]	M318I	43±15	30±5	35.4±0.4	2.98±0.1
		Y320D	60±23	22±8	119±7.8	76.1±6.9
ponatinib	WT		0.2±0.1	0.5±0.3	10.8±0.2	21.8±0.8
	p-nc[2]	L248M	18.0±2.7	2.0±0.6	205±4.2	35.3±1.4

	(from T315)	Y253C	0.4±0.1	2.2±0.3	54.4±0.7	27.7±0.8
		V299L	13±6	8.0±3.0	95.5±0.7	54.5±0.7
	p-c[1] (from I315)	I315M	134±9	4025±595	17.4±0.6	24.2±3.0
bosutinib	WT		5.4±1.2	5.6±0.3	10.8±0.2	21.8±0.8
	p-c[1]	T315I	8700±202	230±25	46.9±0.8	28.6±0.7
	p-nc[2]	L248M	16.7±4.0	37±7.7	205.0±4.2	35.3±1.4

[1]: predicted and clinically occurred mutant

[2]: predicted and clinically not observed mutant

[3]: the measured IC_{50} values for the four drugs are similar to those reported in the literature.

IX. Previous studies on BCR-ABL drug resistance mutations

Supplementary Table 2. A summary of previous studies on BCR-ABL drug resistance mutations

Method	Method class	Drug	Clinical data	Abstract	Drug resistance prediction
Structure-assited point mutation[3]	Experimental	Imatinib	Not required	Extensive experimental mutational analysis of sites that might alter the sensitivity of the ABL kinase to imatinib was performed, demonstrating a broad range of possibilities for clinical resistance	A broad range of possibilities for clinical resistance was predicted and some became evident
Random mutagenesis[4]	Experimental	Imatinib	Not required	To obtain a more comprehensive survey of the amino acid substitutions that confer resistance, an *in vitro* screen of randomly mutagenized BCR-ABL was performed	All of the major mutations previously identified in patients, and numerous others that illuminating novel mechanisms of acquired drug resistance, were recovered. This strategy provided a means of anticipating the drug-resistant mutations amino acid substitutions
Random mutagenesis[5]	Experimental	Nilotinib	Not required	To identify mutations in BCR-ABL that could result in resistance to nilotinib, a cDNA library of BCR-ABL mutants was introduced into Ba/F3 cells followed by selection in	The mutations included 6 known imatinib mesylate–resistant mutations,including T315I, which showed complete resistance to nilotinib. These

				nilotinib	results may predict some of the resistance mutations that will be detected in clinical trials
Imatinib-induced mutagenesis[6]	Experimental	Imatinib	Not required	Imatinib was used to actively and selectively cause sporadic mutations in the BCR-ABL gene	The majority of the mutations of BCR‑ABL were not the clinically observed T315I mutation, suggesting that the T315I mutation may be due to clonal expansion of cells with survival advantages
Molecular docking[7]	Computational	Imatinib, Bosutinib, Nilotinib, Dasatinib Ponatinib	Required	A docking-based computational approach was employed to predict drug sensitivity of 234 point mutations that were reported in chronic myeloid leukemia patients	The computational data generated correlated well with the published experimental. In addition,drug sensitivity profiles for remaining point mutations were presented
Alchemical free-energy calculation[8]	Computational	Imatinib, Bosutinib, Nilotinib, Dasatinib Ponatinib	Required	Alchemical free-energy calculations showed useful accuracy in validating resistance for eight FDA-approved kinase inhibitors across 144 clinically identified point mutations	The method correctly classified mutations as resistant or susceptible with an average of 88% accuracy

X. Supplementary methods

1. Protein heat denaturation

The heat denaturation temperatures of ABL kinase and various mutants were measured using nanoDSF. The nanoDSF (Prometheus NT.48) instrument has become one of the primary techniques to screen for the thermal stability of globular proteins[1]. We used nanoDSF to measure the denaturation temperatures of wild-type ABL kinase and mutants. The temperature range scanned was between 20°C~99°C. The 1 × kinase reaction buffer was used.

Kinase Reaction Buffer:

40mM Tris (pH = 7.5)

20mM $MgCl_2$

0.1mg/mL BSA

2. Protein secondary structure measurement

Protein secondary structures were studied using circular dichroism (wavelength range 190nm~260nm). 1 mm sample cell was used with a protein concentration of 0.2 mg/ml dissolved in PBS buffer at pH 8.0 (20mM K_2HPO_4/KH_2PO_4)

3. Direct binding measurement of ABL kinase and drugs by ITC and MST

We used Isothermal titration calorimetry (ITC) to measure the direct binding constants for imatinib, dasatinib with wt ABL kinase and various mutants. For nilotinib and ponatinib, as the binding enthalpy change is relatively small, we used Microscale thermophoresis (MST) to measure the their binding constants to ABL kinase and mutants.

The ITC200 instrument (Microcal Inc) was for all the ITC studies. The protein was in the sample cell with a concentration of 0.05 mM and the drugs were in the syringe with concentration of 0.5 mM. The buffer used was MES 20mM, NaCl 100mM, pH = 6.5. A total of nineteen 2μL-injections were performed with 4 seconds duration and 120 seconds spacing. The data were analyzed using the Origin software provided by the manufacturer.

MST is based on thermophoresis, which measures the directed motion of molecules in temperature gradients. The Monolith NT.115 MST instrument was used. His-tagged proteins were labeled using the fluorescent dye and kit from the manufacturer. The MST experiments were performed according to the MST manual and the experimental data were analyzed using the software provided by the manufacturer.

4. The half-inhibition constant measurement of drugs and ABL kinase/mutants

The ADP-Glo™ Kinase Assay was used for the half-inhibition constant determination. After the kinase reaction was completed, the reagents that consume the remaining ATP provided by the kit were added (ADP-Glo™ Reagent). After sufficient reaction, the Kinase Detection Reagent from the kit was added.

5. Measuring the catalytic parameters for ABL kinase/mutants using single injection ITC method

Isothermal titration calorimetry (ITC) is a technique that measures the heat released or absorbed during a chemical reaction. This technology is widely used to determine the thermodynamic parameters of biomolecular binding equilibrium. In addition, ITC has been shown to be able to directly measure the kinetic and thermodynamic parameters (k_{cat}, K_M, ΔH) of enzymatic reactions. We have used the single injection method according to the reference[2]. The basic principles are the following:

The rate $\mathbf{R_t}$ of the substrate (ATP) conversion reaction is directly proportional to the power output in the calorimeter cell,

$$\boldsymbol{R_t} = \frac{\boldsymbol{P}}{\boldsymbol{\Delta H \cdot V^0}} \quad \textbf{(1)}$$

where **P** is the power generated by the reaction, **ΔH** is the heat of decomposition of the substrate (ATP), and $\mathbf{V^0}$ is the cell volume. The units of $\mathbf{R_t}$ will be moles/l/sec if **P** is expressed in μcal/sec, **ΔH** in μcal per mole of substrate (ATP), and $\mathbf{V^0}$ in liters, for example.

If Michaelis-Menten kinetics are assumed, then the experimental values for the rate R_t can be expressed as,

$$\boldsymbol{R_t} = \frac{\boldsymbol{k_{cat} \cdot [E]_{totally} \cdot [A]_t}}{\boldsymbol{[A]_t + K_M}} \quad \textbf{(2)}$$

where $\mathbf{k_{cat}}$ is the catalytic rate constant for substrate (ATP) decomposition, $\mathbf{K_M}$ is the Michaelis constant, $\mathbf{[E]_{totally}}$ is the total enzyme concentration, and $\mathbf{[A]_t}$ is the instantaneous concentration of substrate (ATP).

Single injection method. Using this approach, the reaction is initiated by injecting ATP solution from the syringe into the sample cell containing enzyme and reactant solution. The reaction is allowed to complete in the calorimeter cell, and the power **P** is recorded as a function of time **t**. Integration of the excess power **P** associated with the reaction enables **ΔH** to be determined, i.e.,

$$\Delta \mathrm{H} = \frac{\boldsymbol{\int_0^\infty P dt}}{\boldsymbol{[A]_{t=0} \cdot V^0}} \quad \textbf{(3)}$$

where $\mathbf{[A]_{t=0}}$ is the starting substrate (ATP) concentration. Determining **ΔH**, the substrate (ATP) concentration can be determined as a function of time according to the equation:

$$[A]_t = [A]_{t=0} - \frac{\boldsymbol{\int_0^t P dt}}{\boldsymbol{\Delta H \cdot V^0}} \quad \textbf{(4)}$$

After obtaining the time-dependent rate from equation **(1)**, equation **(2)** is used to obtain kinetic parameters by non-linear least squares fitting.

For each experimental run, 400μL of enzyme (10nM) and substrate (1mM) solution and 100μL of ATP (1mM) solution were used. The buffer used was 20 mM Tris-HCl, pH 7.5.

Reference:

1. Lee E, Badr M, Lazic A, Duhr S, Breitsprecher D. Exploring Protein Stability and Aggregation by nanoDSF. *Protein Sci* **25**, 104-104 (2016).

2. Todd MJ, Gomez J. Enzyme kinetics determined using calorimetry: a general assay for enzyme activity? *Anal Biochem* **296**, 179-187 (2001).

3. Corbin AS, Buchdunger E, Pascal F, Druker BJ. Analysis of the structural basis of specificity of inhibition of the ABL kinase by STI571. *J Biol Chem* **277**, 32214-32219 (2002).

4. Azam. M, Latek. RR, Daley. GQ. Mechanisms of Autoinhibition and STI-571/Imatinib Resistance Revealed by Mutagenesis of BCR-ABL. *Cell* **112**, 831–843 (2003).

5. Ray A, Cowan-Jacob SW, Manley PW, Mestan J, Griffin JD. Identification of BCR-ABL point mutations conferring resistance to the ABL kinase inhibitor AMN107 (nilotinib) by a random mutagenesis study. *Blood* **109**, 5011-5015 (2007).

6. Dong Y*, et al.* Semirandom mutagenesis profile of BCRABL during imatinib resistance acquirement in K562 cells. *Mol Med Rep* **16**, 9409-9414 (2017).

7. Kamasani S*, et al.* Computational analysis of ABL kinase mutations allows predicting drug sensitivity against selective kinase inhibitors. *Tumour Biol* **39**, 1010428317701643 (2017).

8. Hauser K*, et al.* Predicting resistance of clinical ABL mutations to targeted kinase inhibitors using alchemical free-energy calculations. *Communications Biology* **1**, 70 (2018).
