# Supplementary material for: A combined computational and experimental strategy identifies mutations conferring resistance to drugs targeting the BCR-ABL fusion protein
Source: Commun Biol. 2020 Jan 9;3:18. doi: 10.1038/s42003-019-0743-5 (PMC6952392; doi:10.1038/s42003-019-0743-5)
Supplement: Supplementary file 2 — Description of Additional Supplementary Files [file 42003_2019_743_MOESM2_ESM.docx]

Data in the "gen_incidence" tab of the supplementary data 1 are used for drawing Figure 2

Data in the "incidence_clinic&prediction" tab of the supplementary data 1 are used for drawing Figure 3

Data in the "KD_normalization" tab of the supplementary data 1 are used for drawing Figure 4

Data in the "IC50_normalization" tab of the supplementary data 1 are used for drawing Figure 5

Data in the "KM&Kcat" and "IC50_normalization" tabs of the supplementary data 1 are used for drawing Figure 6

Data in the "bosutinib&ponatinib" tab of the supplementary data 1 are used for drawing Figure 7

Data in the “Gefitinib” tab of the supplementary data 1 are used for drawing Figure 8

P.S. Figure 1 is a data-free flow chart, drawn with Microsoft Visio.
